# Acidic leucine-rich nuclear phosphoprotein-32A (ANP32A) association with lymph node metastasis predicts poor survival in oral squamous cell carcinoma patients

**DOI:** 10.18632/oncotarget.7681

**Published:** 2016-02-24

**Authors:** Bharath Kumar Velmurugan, Kun-Tu Yeh, Chien-Hung Lee, Shu-Hui Lin, Mei-Chung Chin, Shang-Lun Chiang, Zhi-Hong Wang, Chun-Hung Hua, Ming-Hsui Tsai, Jan-Gowth Chang, Ying-Chin Ko

**Affiliations:** ^1^ Environment-Omics-Diseases Research, China Medical University Hospital, Taichung, Taiwan; ^2^ Department of Pathology, Changhua Christian Hospital, Changhua, Taiwan; ^3^ School of Medicine, Chung Shan Medical University, Taichung, Taiwan; ^4^ Department of Public Health, College of Health Science, Kaohsiung Medical University, Kaohsiung, Taiwan; ^5^ Department of Health Risk Management, College of Public Health, China Medical University, Taichung, Taiwan; ^6^ Department of Otorhinolaryngology, China Medical University Hospital, Taichung, Taiwan; ^7^ Department of Laboratory Medicine, China Medical University Hospital, Taichung, Taiwan; ^8^ Graduate Institute of Clinical Medical Science, China Medical University, Taichung, Taiwan

**Keywords:** OSCC, Claudlin-1, ANP32A, Slug, EMT, Pathology Section

## Abstract

Acidic leucine-rich nuclear phosphoprotein-32A (ANP32A) is a multifunctional protein aberrantly expressed in various types of cancers. However, its expression pattern and clinical significance in oral squamous cell carcinoma (OSCC) remains unclear. In this study, we immunohistochemically investigated the expression pattern of ANP32A in 259 OSCC patients and the results were correlated with clinicopathological factors using Allred, Klein and Immunoreactive scoring (IRS) system. Our data indicated that high expression of ANP32A was significantly associated with N stage and tumor differentiation status in OSCC patients. High ANP32A expression with N2/N3 stage had an increased mortality risk than low ANP32A expressing OSCC patients with N0/N1 stage. Functional studies revealed that knockdown of ANP32A significantly decreased the migration and invasion ability thereby concomitantly increasing E-cadherin and decreasing Slug, Claudin-1 and Vimentin expression *in vitro*. These results suggest that ANP32A is commonly increased in oral squamous cell carcinoma and ANP32A protein could act as a potential biomarker for prognosis assessment of oral cancer patients with lymph node metastasis.

## INTRODUCTION

Oral squamous cell carcinoma (OSCC) is the sixth leading cause of cancer death in the worldwide (The age- standardized per 100,000 GLOBOCAN 2008, IARC); in Taiwan more than 6500 new cases of oral and pharyngeal cancer are diagnosed annually (7-8% of all cancers) [1]. Despite of the improved diagnostic technique its prognosis remains poor; indeed, the 5-year survival rate remained around 50-55% over the past several decades [2-4]. Therefore, it is of important to identify the molecular markers that are associated with OSCC malignancy; which may further improve the clinical management and therapeutic development [5, 6].

Acidic leucine rich nuclear phosphoprotein-32A (ANP32A) is nuclear phosphorproteins that are expressed in normal tissues as well as in breast, pancreas, and prostate cancers [7-9]. ANP32A (pp32), possess variety of cellular functions such as cell differentiation, transcription, apoptosis and cell cycle progression [8, 10-12]. In pancreatic adenocarcinoma expression of ANP32A was well correlated with their differentiation status. This well differentiated tumors showed normal expression of ANP32A, whereas reduced or absent in poorly differentiated tumors [13]. Most of the studies have found that, ANP32A function as a tumor suppressor protein [9, 14], surprisingly increased expression was found in hepatocellular carcinoma [15], colorectal cancer [16], pancreatic tumor [17] and liver cancer [15]. However, whether the aberrant expression of ANP32A in OSCC is associated with malignancy, metastasis, or prognosis remains unknown.

In this present study we determined the expression pattern of ANP32A in oral squamous cell carcinoma and its correlation with clinicopathological features, including the overall survival of OSCC patients. Additionally, we examined the functional role of ANP32A on EMT pathway using highly invasive oral cancer cell- HSC-3, *in vitro*

## RESULTS

### Expression profile of ANP32A in tumor and normal tissues of OSCC patients

**Figure 1:**
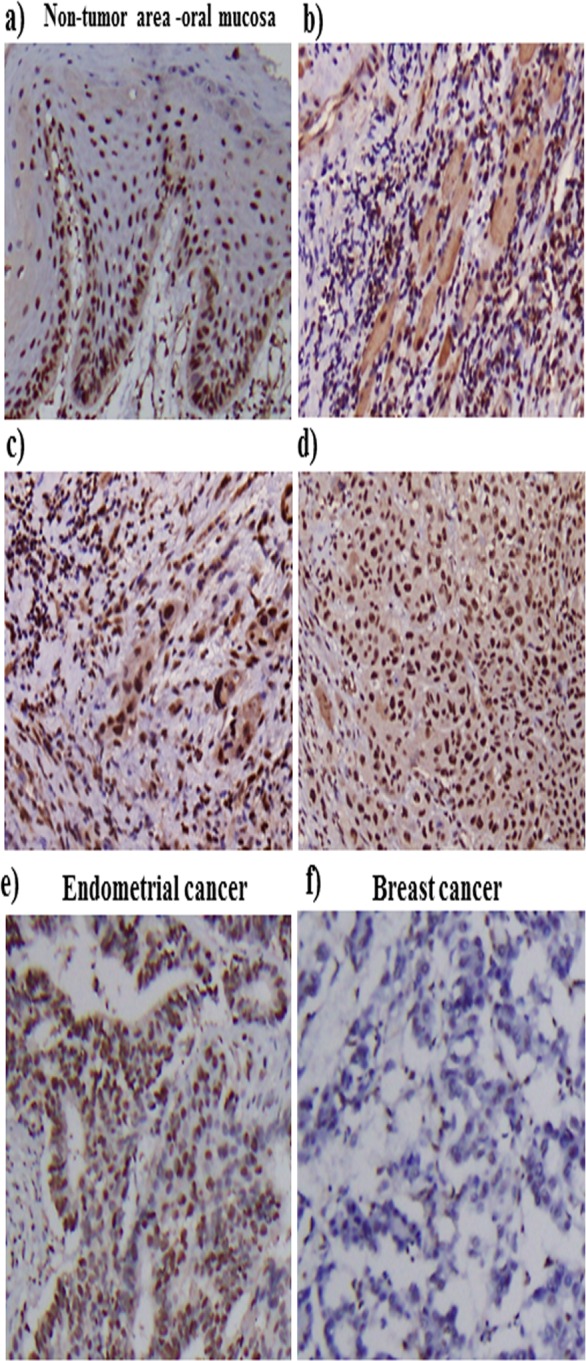
Higher expression of AN32A in OSCC patients ANP32A expression was detected in oral cancer patients and in non-tumor tissues by performing immunohistochemistry. **a.** ANP32A expression in the non-tumor areas of oral mucosa. **b.**-**d.** Different staining intensities of ANP32A in OSCC tissues. The scoring criteria for ANP32A immunoreactivity are described in the Materials and methods section. The cellular staining was classified using a scale of (±, 1+, 2+) as follows: ± weak staining **b.**, 1+ moderate staining **c.**, 2+ strong staining **d.**.**e.** & **f.** Endometrial and breast cancer was used as a positive and negative control respectively. Representative images show the consistent expression of ANP32A. Left panel: magnification×100. Right panel: magnification ×400.

To study the prevalence of ANP32A in OSCC patients, at first we examined the expression pattern of ANP32A using immunohistochemistry on the tumor tissues and adjacent non-tumor tissues from 259 patients with OSCC (Figure 1). ANP32A was observed in both nucleus and cytoplasm of OSCC cells and in non-tumor tissues. Compared to the non-tumor tissues, the level of ANP32A staining was higher in the OSCC cells. ANP32A staining was scored as “1+” if the staining intensity was lower than the staining intensity of the normal oral squamous mucosa. The staining was scored as “2+” if the staining intensity in the neoplasm matched the staining intensity of the normal oral squamous mucosa. A score of “±” reflected a lack of ANP32A immunoreactivity compared with the staining pattern of normal oral squamous mucosa. In a total of 259 OSCC cases, ANP32A expression was negative or weak positive (±) in 114 cases, moderately positive in 105 cases (with IHC scores of 1+) and strongly positive in 40 cases (with IHC scores of 2+). To determine the antibody specificity we used endometrial cancer as a positive control and breast cancer as a negative control (Figure 1e & 1f).

### Association between ANP32A and clinicopathologic factors

The demographic and clinicopathologic characteristics for OSCC patients were shown in Table 1. A predominant proportion of oral malignant cases were male (95%), as compared to female (5%). Among the cancer patients, 44% and 22.7% were found to have tumor site of stage III/IV and lymph node metastasis of N2/N3, respectively, and 84.2% were moderate or poor histological grade. A total of 98 OSCC patients (37.8%) received surgery therapy and 161 patients (62.2%) were treated with radiotherapy or/and chemotherapy.

**Table 1:** Demographic and characteristics among oral cancer patients

Factors	No	%
Gender		
Female	13	5.0
Male	246	95.0
Age, year		
≤49	79	30.5
50-59	95	36.7
60-69	57	22.0
≥70	28	10.8
T (tumor size)		
I	64	24.7
II	81	31.3
III	22	8.5
IV	92	35.5
N (lymph node)		
N0	167	64.5
N1	33	12.7
N2	55	21.2
N3	4	1.5
M (metastasis)		
No	257	99.6
Yes	1	0.4
AJCC cancer stage		
I	50	19.3
II	54	20.9
III	33	12.7
IV	122	47.1
Histological grade		
Well	41	15.8
Moderate	211	81.5
Poor	7	2.7
Clinical therapy		
Surgery	98	37.8
Radiotherapy	96	37.1
Chemotherapy	1	0.4
Chemoradiotherapy	64	24.7

The association between clinicopathologic factors and ANP32A expression by different IHC scoring systems in OSCC patients was presented in Table 2. Patients with a high level of ANP32A expression defined by Allred and IRS scoring systems were respectively found to have a 2.2-fold (95% CI, 1.2-4.0) and 2.0-fold (95% CI, 1.1-3.6) risk of contracting N2/N3 of lymph node metastasis. Likewise, a higher ANP32A expression was related to moderate/poor tumor differentiation in these two scoring systems (adjusted odds ratios, aOR = 2.2, *P* < 0.05, in both systems). No significant relationship between ANP32A expression and other clinicopathologic parameters were identified. However, using Klein scoring system no any significant association was found between ANP32A expression and pathological parameters. This results are in correlation with Fedchenko N *et al.* [18] hypothesis that, each IHC marker should have an individual scoring systems. Thus we chose to use IRS and Allred scoring system to interpret and further analysis our results.

**Table 2:** Clinicopathologic factors associated with ANP32A expression by different IHC scoring systems among OSCC patients

Factors	Allred				Klein				IRS					
	Low No (n = 151)	High No. (n = 108)	aOR^a^	(95% CI)	Low No (n = 151)	High No. (n = 108)	aOR^a^	(95% CI)	Low No. (n = 159)	High No. (n = 100)	aOR^a^	(95% CI)
T (tumor size)												
I/II	83	62	1.0		126	19	1.0		87	58	1.0	
III/IV	68	46	0.9	(0.6-1.5)	98	16	1.1	(0.6-2.4)	72	42	0.9	(0.5-1.5)
N (lymph node)												
N0/N1	125	75	1.0		176	24	1.0		130	70	1.0	
N2/N3	26	33	2.2	(1.2-4.0)	48	11	1.8	(0.8-4.0)	29	30	2.0	(1.1-3.6)
M (metastasis)												
No	150	107	1.0		222	35	1.0		158	99	1.0	
Yes	0	1	-	ND	1	0	-	ND	0	1	-	ND
AJCC cancer stage												
I	32	18	1.0		46	4	1.0		33	17	1.0	
II	30	24	1.5	(0.7-3.4)	46	8	2.0	(0.6-7.4)	33	21	1.3	(0.6-3.0)
III	20	13	1.3	(0.5-3.3)	28	5	2.3	(0.6-9.7)	20	13	1.5	(0.6-3.8)
IV	69	53	1.5	(0.7-3.0)	104	18	2.2	(0.7-6.9)	73	49	1.4	(0.7-2.9)
Histological grade												
Well	30	11	1.0		37	4	1.0		31	10	1.0	
Moderate/Poor	121	97	2.2	(1.0-4.7)*	187	31	1.5	(0.5-4.7)	128	90	2.2	(1.0-4.7)*

a Adjusted odds ratio (aOR) was controlled for gender and age.

* *P* < 0.05; ND, not determined.

### ANP32A expression and survival

The effects of clinicopathologic factor and ANP32A expression on mortality in the OSCC patient cohort were shown in Table 3. The mean follow-up time for this cohort was 5.4 year (SD, 3.9 years). The mortality density for patients with N2/N3 of lymph node metastasis and moderate/poor tumor differentiation was 23.5 and 9.0 per 100 people-years, respectively. A higher mortality risk was observed to be related to a higher level of tumor size, lymph node metastasis, tumor stage, tumor differentiation and chemotherapy/radiotherapy (aHR = 2.0, 3.0, 2.7, 2.9 and 3.7, respectively). The independent mortality risk for patients with high ANP32A expression was non-significant as compared with those with low expression, regardless the use of Allred and IRS scoring systems (both *P* > 0.05).

**Figure 2:**
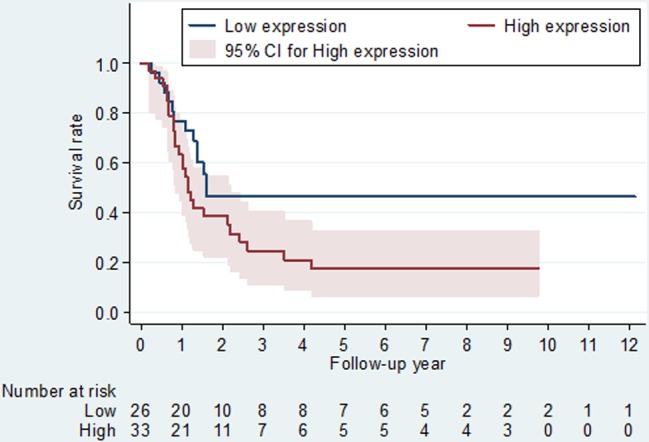
Kaplan-Meier survival curves and at-risk tables associated with ANP32A expression defined by the Allred scoring system among oral cancer patients with N2+N3 lymph node stage *P*-values obtained from log-rank tests for the homogeneity of Kaplan-Meier curves between high and low ANP32A expressions was 0.049.

Because ANP32A expression was associated with lymph node metastasis and tumor differentiation, we further evaluated the joint effect of ANP32A expression and the two pathological factors on mortality. The combined Kaplan-Meier mortality curves associated with lymph node stage and ANP32A expression using Allred and IRS scoring systems were significantly heterogeneous (*P* < 0.001 and 0.002, respectively, Supplementary Figure 1 and 2). Similar findings were found in the joint mortality curves in relation to tumor differentiation and ANP32A expression (Supplementary Figure 3 and 4). As compared to patients with N0/N1 stage and low ANP32A expression, the mortality hazard risk was multiplicatively enhanced among patients with N2/N3 stage and high ANP32A expression (aHR = 1.8, 95% CI, 1.1-3.8; *P* = 0.008 for multiplicative interaction, Table 3). Focusing on OSCC patients with N2/N3 stage of lymph node metastasis (*N* = 59), patients with Allred defined high level of ANP32A expression was observed to have a worse survival curves (*P* = 0.049 for log-rank tests, Figure 2) and a 2.1-fold higher hazard risk (95% CI, 1.0-4.2) than patients with low ANP32A expression.

**Table 3:** The effect of clinicopathologic factor and ANP32A expression on mortality density and adjusted hazard ratio (aHR) among OSCC patients

Factors	No. of patient	Follow-up (person-year)	No. of death	Mortality density^a^	aHR^b^	(95% CI)
**Overall mortality from primary malignancy to death**						
T classification						
I/II	145	890.9	46	5.2	1.0	
III/IV	114	496.4	61	12.3	2.0	(1.4-3.0)
N classification						
N0/N1	200	1221.4	68	5.6	1.0	
N2/N3	59	165.8	39	23.5	3.0	(2.0-4.5)
AJCC tumor stage						
I/II	104	717.6	26	3.6	1.0	
III/IV	155	669.7	81	12.1	2.7	(1.7-4.3)
Tumor differentiation						
Well	41	283.5	8	2.8	1.0	
Moderate/Poor	218	1103.8	99	9.0	2.9	(1.4-6.1)
Clinical therapy						
Surgery	98	690.1	20	2.9	1.0	
Chemotherapy/Radiotherapy	161	697.1	87	12.5	3.7	(2.2-6.0)
Allred						
Low	151	816.8	59	7.2	1.0	
High	108	570.4	48	8.4	1.2	(0.8-1.8)
IRS						
Low	159	870.1	61	7.0	1.0	
High	100	517.1	46	8.9	1.3	(0.9-1.9)
Combined effect						
N stage/Allred^c^						
N0+N1/Low	125	732.4	46	6.3	1.0	
N0+N1//High	75	489.0	22	4.5	0.6	(0.4-1.1)
N2+N3/Low	26	84.4	13	15.4	0.9	(0.5-1.8)
N2+N3/High	33	81.4	26	32.0	1.8	(1.1-3.8)^d^
N stage/IRS^c^						
N0+N1/Low	130	783.4	45	5.7	1.0	
N0+N1//High	70	438.0	23	5.3	0.8	(0.4-1.3)
N2+N3/Low	29	86.7	16	18.5	1.2	(0.6-2.2)
N2+N3/High	30	79.1	23	29.1	1.8	(1.0-3.1)
**Overall mortality from malignancy with N2+N3 stage to death**						
Allred^c^						
Low	26	84.4	13	15.4	1.0	
High	33	81.4	26	32.0	2.1	(1.0-4.2)
IRS^c^						
Low	29	86.7	16	18.5	1.0	
High	30	79.1	23	29.1	1.6	(0.8-3.1)

a Mortaility density was displayed as per 100 people-years.

b aHR was adjusted for gender and age.

c HR was adjusted for gender, age, tumor differentiation, tumor stage and therapy method.

d Significant multiplicative-scale interaction between N2+N3 stage and high expression on mortality risk was identified (*P* for interaction, 0.008).

### ANP32A protein expression in OSCC-derived cell lines

**Figure 3:**
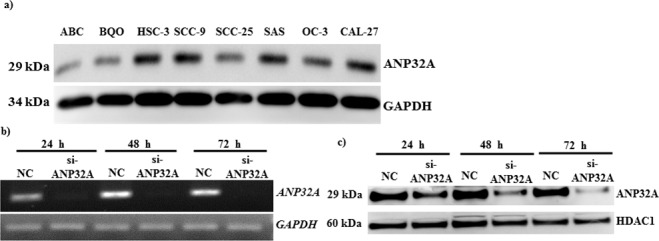
Immunoblot analysis of ANP32A expression **a.** Oral cancer cells were analyzed for ANP32A expression; GAPDH was used as a loading control. HSC-3 cells were transfected with siControl (siCtrl) or si-ANP32A for different time points and analyzed for ANP32A **b.** mRNA expression by RT-PCR, and c) protein expression by western blotting. GAPDH and HDAC1 were used as an internal control, respectively.

To investigate ANP32A function, we examined ANP32A levels in a panel of human OSCC cell lines. As shown in Figure 3a, ANP32A was found to over express to varying degrees in oral cancer cells. A high metastatic human tongue cancer cell line HSC-3 that showed a high ANP32A expression was chosen to do the further experiments. To evaluate the significance of ANP32A in high metastatic cells, we transiently transfected si-ANP32A for 72 h and then analyzed for its mRNA and protein expression. The amount of ANP32A mRNA and protein levels was reduced greatly compared with the negative control cells (Figure 3b & 3c). Our results showed that 48 h time duration was sufficient to block the mRNA and protein expression of ANP32A. Thus we chose 48 h time point to do the downstream experiment.

### Effect of ANP32A depletion on EMT

**Figure 4:**
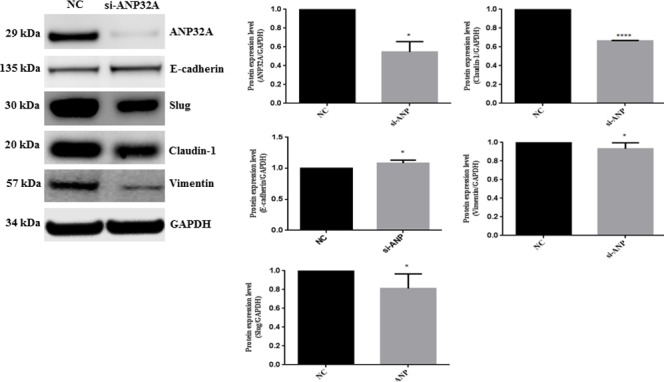
Knock down of endogenous ANP32A expression altered EMT proteins- E-cadherin, Slug, Claudlin-1 and Vimentin expression GAPDH was used as a loading control. Densitometry values are represented in bar chart. Data mean values ± SD. The asterisks indicate a significant (*, *P* < 0.05, **P < 0.01) difference between the si Control (siCtrl) and si-ANP32A treated groups.

Because the clinical significance of ANP32A overexpression was linked to lymphnode metastasis, we evaluated the expression difference of several epithelial-mesenchymal transition (EMT) markers between ANP32A knockdown cells and control cells. We found a significant decrease in Slug, Claudin-1 and Vimentin while an epithelial marker E-cadherin was increased in HSC-3 (Figure 4).

### Reduced ANP32A expression decreases OSCC cell migration and invasion

**Figure 5:**
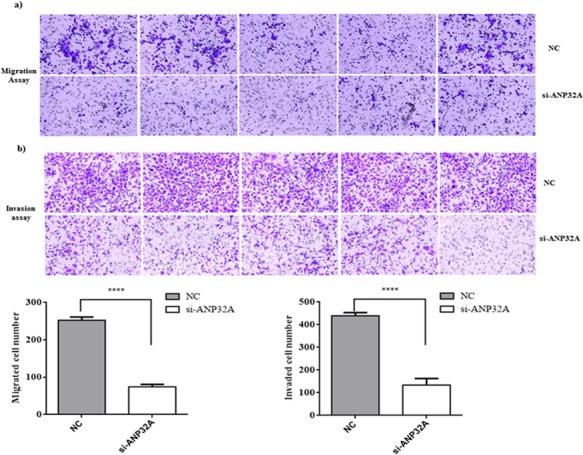
Knockdown of ANP32A reduces OSCC cell migration and invasion HSC-3 cells were transiently transfected with ANP32A-specific siRNA, a nonspecific siRNA (negative control). Representative photos of **a.** migration assay and **b.**matrigel invasion assay using Boyden chamber. Quantification of relative numbers of migrated and invaded cells represents average counts from five fields of view. Data mean values ± SD. ****P < 0.0001.

HSC-3 cells were treated with si-ANP32A for 48h and then seeded in Boyden chamber with or without matrigel for 24 h to deter­mine whether ANP32A blockade could decrease their migration and invasion potential. ANP32A inhibition dramatically decreased the migration ability and number of migrated cells when compared to the negative control group (Figure 5a). We further examined the invasive ability in ANP32A inhibited group; ANP32A inhibition remarkably decreased the number of invaded cells compared with the negative control (Figure 5b). These findings suggest ANP32A expression may involve in EMT progression to promote invasion and metasta­sis of HSC-3.

## DISCUSSION

It has been long recognized that ANP32A function as a tumor suppressor in several human cancers, including pancreatic [9, 13, 17] and breast cancer [19, 20]. In Pancreatic cancer, their expression level was highly associated with tumor differentiation [17]. However, no any information is available about ANP32A expression and its prognostic significance in OSCC. In this present study we identified and quantified ANP32A protein expression in OSCC tissue and normal marginal tissues by IHC. We found that nucleus expression of ANP32A was abundantly found in all the samples (100%) and positive cytoplasmic expression was found in 73.7% of patients. Our findings also showed that high ANP32A protein level was associated with a higher level of lymph node metastasis and tumor differentiation, but not with TM classification and tumor stage.

The presence of lymph node metastasis at the time of diagnosis is one of the most important prognostic factors in OSCC [21]. Therefore, an efficient biomarker is essential for earlier determination of lymph node metastasis that may help physician to have their therapy options [22]. In this present study, we found that lymph node metastasis (N2/N3) patients with high ANP32A expression had a worst prognosis at a 10 year follow up. To our knowledge, this is the first study that shows the correlation between ANP32A expression by IHC and lymph node metastasis and overall survival among OSCC patients using Allred and IRS system. However, no any significant association was found between ANP32A expression and clinical characteristics. From our study it is evident that, Allred and IRS can be used as a golden standard system to evaluate ANP32A expression in various other cancers. Taken together, these results indicate that ANP32A could serve as a potential biomarker of lymph node metastasis in OSCC.

Earlier studies suggested a role for ANP32A expression as a prognostic biomarker in pancreatic cancer. Brody *et al.* correlated ANP32A expression in well-differentiated adenocarcinomas, and in a subset of moderately differentiated adenocarcinomas. ANP32A was absent or reduced in poorly differentiated tumors and in intraductal papillary mucinous neoplasms with moderate dysplasia [13]. However, it has been reported that ANP32A overexpression was not significantly correlated with pathological parameter in cancers like pancreatic and non-small cell lung cancer. Thus, ANP32A expression seems to behave in a tissue specific manner.

In this present study, ANP32A expression was significantly associated with oral cancer tumor invasiveness and lymphnode metastasis. Therefore, ANP32A-expressing cells have clinical implication for the development and progression of OSCC. The level of ANP32A expression may be considered to use as a biomarker for malignancy of OSCC and a predictor of patient prognosis. We further investigated whether ANP32A over expression increased the invasive potential of oral carcinoma cell lines. A highly malignant and invasive oral cancer HSC-3cell isolated from metastatic tumors in a lymph node was chosen to perform the *in vitro* study [23, 24]. Abrogating ANP32A expression in highly invasive oral cancer cell decreases its metastasis and invasive levels. Our findings suggest that ANP32A expression increases as cancer cells progress toward malignant phenotype. EMT is critical in tumor progression and metastasis formation and it is related with worse prognosis for cancer patients [25, 26]. D uring EMT transformation epithelial markers (such as E-cadherin, desmoplakin, cytokeratins, claudins, occluding, and beta-catenin) were decreased and the mesenchymal markers (such as vimentin, n-cadherin, fibronectin, and snail-1/2) were increased [27-30]. Recent studies have shown that claudin-1is aberrantly expressed in diverse types of human cancers, including OSCCs [31]. In this study, we found that knockdown of ANP32A decreased Snail, Vimentin, Claudin-1 and increased the level of E-cadherin expression *in vitro*. We found highly significant association between ANP32A and Claudin-1expression compared with other EMT marker. These results support the notion that ANP32A increased cancer cell invasion through EMT pathway.

In conclusion we report here for the first time, that ANP32A is aberrantly expressed in OSCC tissues and in OSCC-derived cell lines. High ANP32A expression was associated with high stage of lymphnode metastasis at cancer diagnosis, and the mortality hazard risk was multiplicatively enhanced among N2/N3 patients with high ANP32A expression. Our *in vitro* results further showed that, knockdown of ANP32A in oral cancer cells reduced its invasive and metastatic ability. However, further investigations are necessary to understand their molecular role in oral cancer progression and metastasis.

## MATERIALS AND METHODS

### Participants and clinical tissues

To evaluate the relationship of ANP32A expression with clinical/pathological factors and survival, a total of 259 oral cancer tissue samples from tissue blocks were obtained from the Changhua Christian Hospital. The tumors were classified according to the International Union against Cancer TNM classification system [32]. Only specimens without any treatment were selected for this study to avoid possible influences of the treatment modality. The present study was approved by the ethics committee of China medical University Hospital and Changhua Christian Hospital according to the declaration of Helsinki. Informed consent was obtained from all patients for this study.

### OSCC cell lines

ABC and BQO primary oral cancer cells were originally established in our lab. OC-3 cell were obtained from Dr. S.C. Lin (National Yang Ming University, Taipei, Taiwan) [33] and oral cancer cell lines, SAS, HSC-3, SCC-9, SCC-25, SAS and CAL-27 were purchased from the Bio-resource Collection and Research Center, Taiwan and maintained in DMEM- F12 (Invitrogen; Carlsbad, CA) medium supplemented with 10% fetal bovine serum. OC-3 cells were cultured in a 1:2 mixture of DMEM-F12 and KSFM with 10% FBS. All the cells are incubated in a humidified 5% CO_2_ atmosphere at 37°C.

### Tissue microarray

Formalin-fixed, paraffin-embedded block of cancer tissues are used in this experiment. The tissue cores were arranged into new paraffin blocks by using a fine steel needle to create the tissue microarrays. Cancer tissues were sliced to 4 μm thickness and then hematoxylin and eosin staining was performed to confirm the presence of the original cancers as indicated by morphologically representative areas [34].

### Immunohistochemical staining and scoring

The Immunohistochemical staining methods have been described previously [34]. Tissue sections were deparaffinized and rehydrated using routine techniques. Endogenous peroxidase activity was blocked with 3% H_2_O_2_ in methanol, hydrated with gradient alcohol and phosphate-buffered saline solution, and incubated in 10 mmol/L citrate buffer (pH 6.0). The sections were incubated with ANP32A rabbit polyclonal antibody (Abcam, Cambridge, United Kingdom) in room temperature for 20 mins. After washing three times with PBS, the sections were incubated with appropriate peroxidase-labelled secondary antibodies for 30 min at room temperature. The sections were washed three times with PBS and then labelled by diaminobenzidine and counterstained with Mayer's haematoxylin, dehydrated and mounted.

Each tumor was given a score according to the intensity of the nuclear/cytoplasmic staining: Negative staining and weak positive staining, ±; moderate staining, 1+; and strong staining, 2+. Staining intensity was confirmed by 2 pathologists. For Allred scoring system, the cut-off score ≤ 5 and > 5 were defined as low and high expression level, respectively. For Klein scoring system and immunoreactive scoring (IRS) system, the cut-off score ≤ 3 and > 3 were defined as low and high expression level, respectively.

### Small interfering RNA transfections

Double-stranded small interfering RNA (siRNA) oligonucleotides (25 pmol per 6 cm plate; ANP32A: 5′-UUUGGUAAGUUUGCGAUUGag-3′; CY3 dye labeled pre-miR Negative Control 1 (Ambion, Foster City, CA, AM17120), both were transfected with RNAiMAX reagent (Invitrogen, Carlsbad, CA) according to the manufacturer's instructions.

### Migration and invasion assay

Invasion assay was performed as mentioned earlier [35]. Cell migration and invasion was performed at a 24-well Transwell chamber with a pore size of 8 *μ*m (Corning, Bedford, MA, USA). The insert was coated with 100 μl Matrigel (dilution at 1: 2; Corning, Bedford, MA, USA). HSC-3 cells were trypsinised after transfection with control or ANP32A siRNA for 48h and transferred to the upper chamber with or without Matrigel in 100 μl of serum free medium containing cells and incubated for 24 h. The lower chamber was filled with complete media. The membranes were fixed and stained using 0.1% crystal violet.

### Western blot analysis

Immunoblotting was performed with primary antibodies against ANP32A (Abcam, Cambridge, United Kingdom), E-cadherin, Slug, Vimentin and Claudin-1 (Cell Signaling Technology, Danvers, MA, USA). GAPDH (Millipore, Billerica, MA, USA), HDAC1 (Santa Cruz Biotechnology, Santa Cruz, CA, USA) was used as a loading control. The signal was visualized with super enhanced chemiluminescence (ECL) detection reagent (Millipore, Billerica, MA, USA).

### RNA isolation and real-time PCR

Total RNA was isolated from HSC-3 cells transfected with negative control or ANP32A siRNAs, using the Trizol reagent (Invitrogen, Burlington, ON, USA) according to the manufacturer's instruction. Isolated RNA was used as a template for the reverse transcription reaction (Invitrogen, Carlsbad, CA). The ANP32A sense primer was 5′-GCCTCACCTCAATCGCAAAC-3′, and the antisense primer was 5′-CCCCTGAGACTCTGTTATCGC-3′. For GAPDH gene, the sense primer was 5′-GAGAAGGCTGGGGCTCATTT-3′ and the antisense primer was 5′-AGTGATGGCATGGACTGTGG-3′.

### Statistical analysis

Proportions were used to describe the distribution of demographic and clinicopathologic characteristics in the patient cohort with oral squamous cell carcinoma. Multivariate logistic regression models were employed to evaluate the association between clinicopathologic factors and ANP32A expression defined by Allred, Klein and IRS scoring systems, respectively. We used Kaplan-Meier curves to assess survival of the patients having diverse ANP32A expressionover time, with differences appraised using the log-rank test for equality of survivor functions. The adjusted hazard ratio (aHR) and corresponding 95% confidence intervals (CIs) obtained from the multivariate Cox proportional-hazards regression models wereemployed to determine the predictive effect of ANP32A expression on survival. Overall survival was separately computed for all patients and for the patients with N2 or N3 lymph node stages. The two overall survivals were calculated from the date of primary malignancy diagnosis and the date of diagnosis of N2 or N3 lymph node stages to the last follow-up (censored) or death from malignancy (event), respectively. Gender and age, and where appropriate, tumor stage, tumor differentiation and therapy scheme were included in the multivariate Cox model as the covariates. All *P* values were two-tailed, and a *P*value of < 0.05 was considered statistically significant.

## SUPPLEMENTARY FIGURES AND TABLES


