# Supplementary material for: Acidic leucine-rich nuclear phosphoprotein-32A (ANP32A) association with lymph node metastasis predicts poor survival in oral squamous cell carcinoma patients
Source: Oncotarget. 2016 Feb 24;7(10):10879–90. doi: 10.18632/oncotarget.7681 (PMC4905446; doi:10.18632/oncotarget.7681)
Supplement: Supplementary file 1 [file oncotarget-07-10879-s001.pdf]

Acidic leucine-rich nuclear phosphoprotein-32A (ANP32A) association with lymph node metastasis predicts poor survival in oral squamous cell carcinoma patients

Supplementary Material

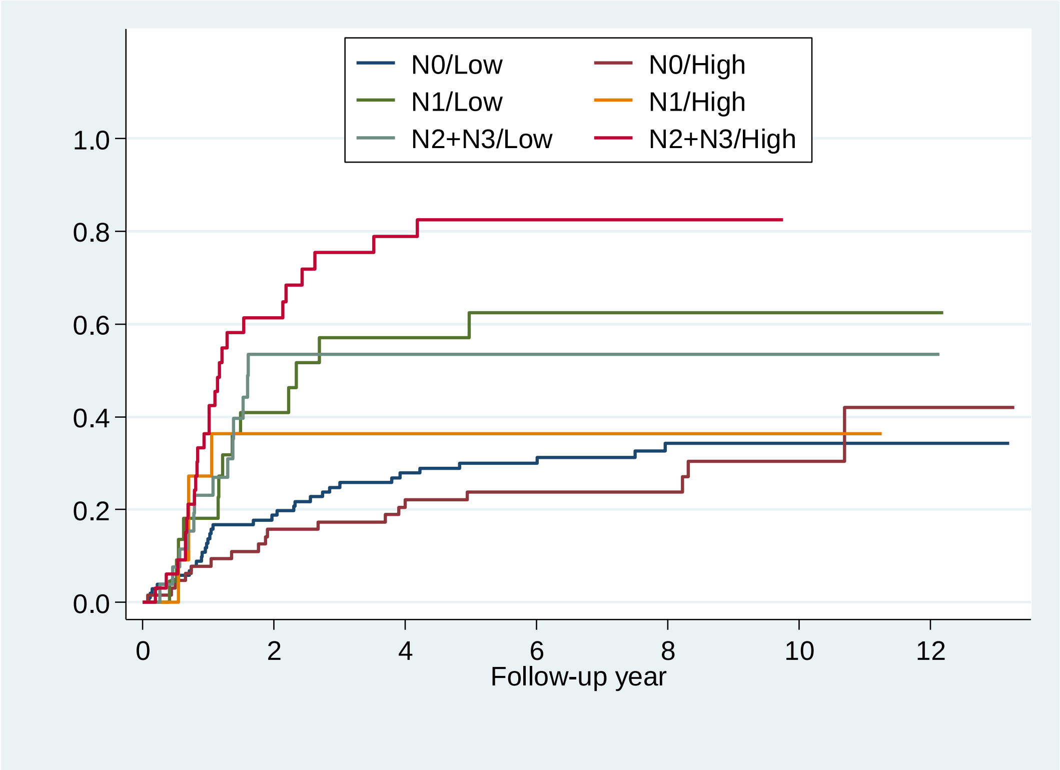

Supplementary Figure 1: Kaplan-Meier mortality curves associated with N stage (N0, N1 and N2+N3) and Allred expression (Low and High) in oral cancer patients.
Note: *P*-values obtained from log-rank tests adjusted for gender, age, clinical therapy method for the homogeneity of Kaplan-Meier curves between combined groups of N stage and Allred expressions was <0.001.

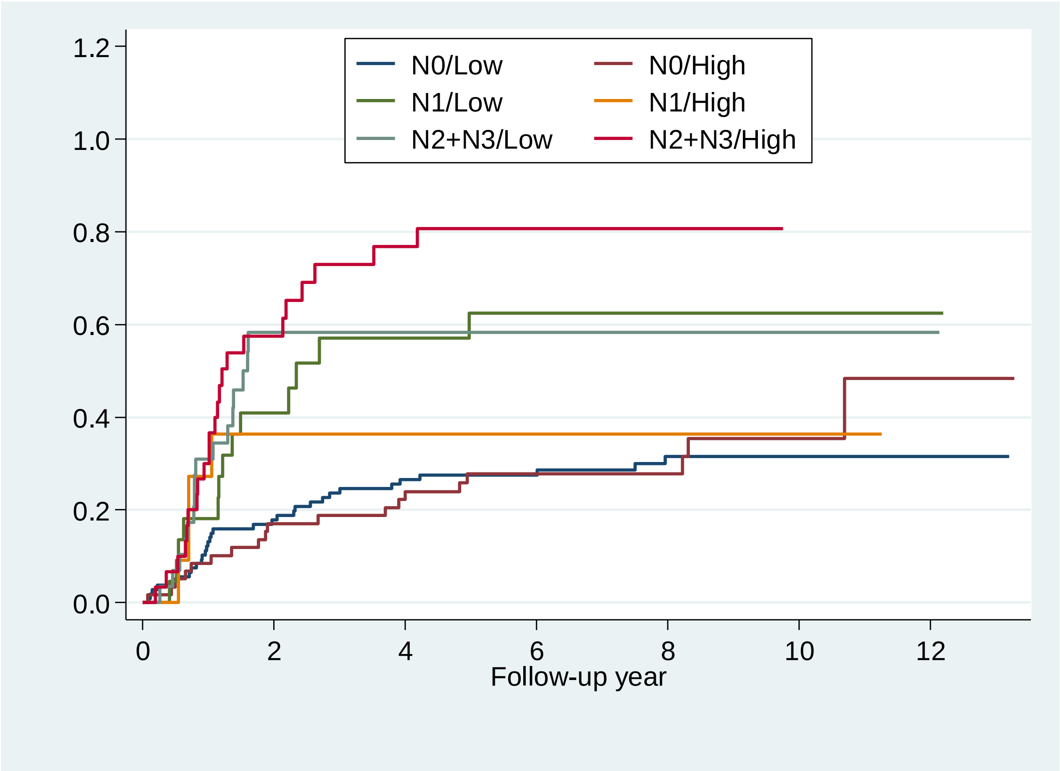

Supplementary Figure 2: Kaplan-Meier mortality curves associated with N stage (N0, N1 and N2+N3) and IRS expression (Low and High) in oral cancer patients.
Note: *P*-values obtained from log-rank tests adjusted for gender, age and clinical therapy method for the homogeneity of Kaplan-Meier curves between combined groups of N stage and IRS expressions was 0.002.

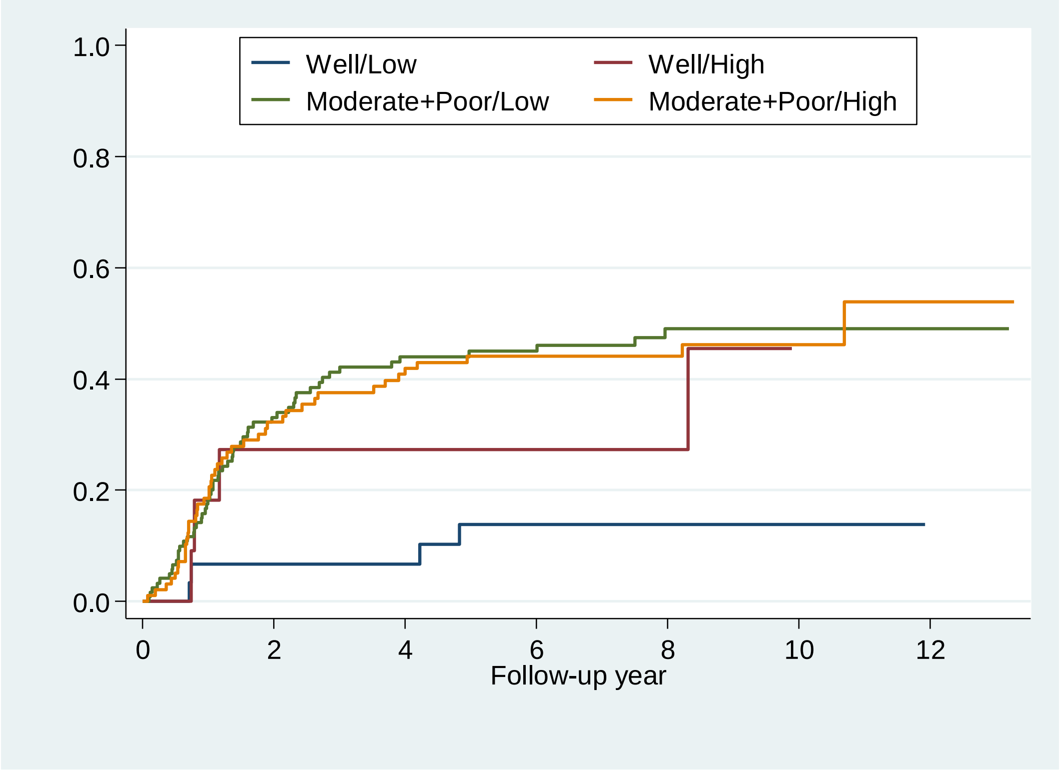

Supplementary Figure 3: Kaplan-Meier mortality curves associated with tumor differentiation (Well and Moderate+Poor) and Allred expression (Low and High) in oral cancer patients.

Note: *P*-values obtained from log-rank tests adjusted for gender, age, clinical therapy method for the homogeneity of Kaplan-Meier curves between combined groups of tumor differentiation and Allred expressions was 0.080.

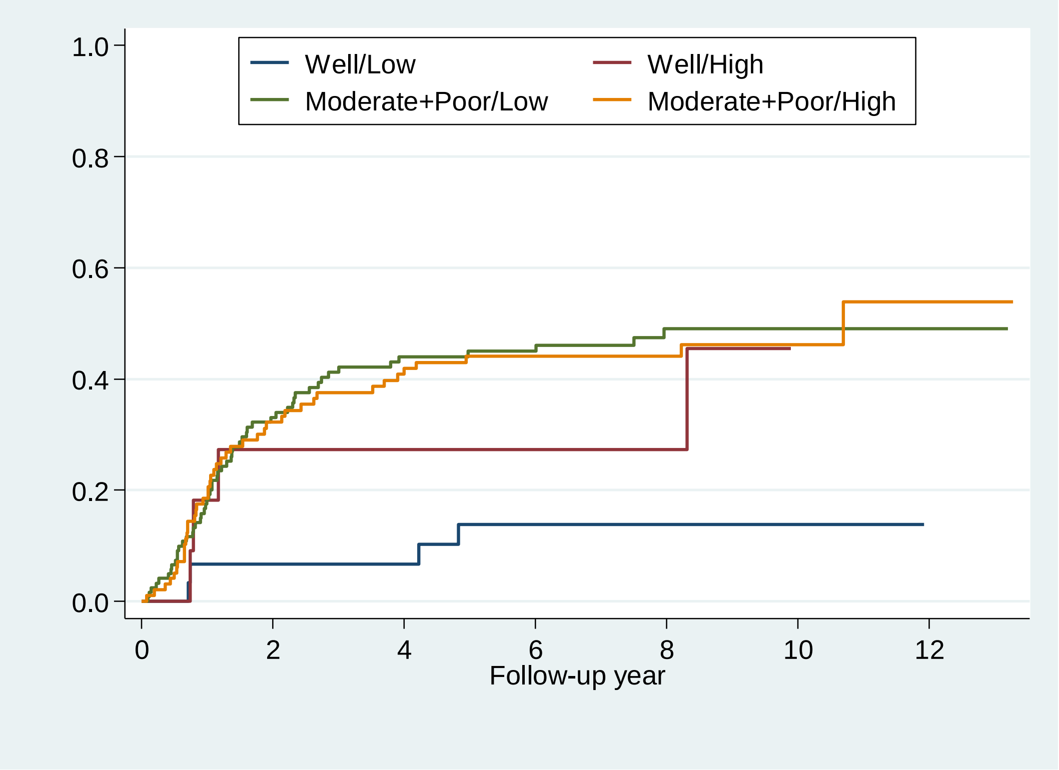

Supplementary figure 4. Kaplan-Meier mortality curves associated with tumor differentiation (Well and Moderate+Poor) and IRS expression (Low and High) in oral cancer patients.

Note: *P*-values obtained from log-rank tests adjusted for gender, age, clinical therapy method for the homogeneity of Kaplan-Meier curves between combined groups of tumor differentiation and IRS expressions was 0.083.
